# Wellness, Resilience, and Burnout Among Pediatric Rheumatology Fellows: A Narrative Medicine Pilot Intervention

**DOI:** 10.3390/healthcare14132025

**Published:** 2026-07-07

**Authors:** Aviya Lanis Levy, Jacob Spitznagle, Kathryn Cook, Sheetal S. Vora, Kimberly Hays, Kara Murphy Schmidt, Courtney Wells, Aryn Bartley, Nandini Moorthy

**Affiliations:** 1 Rady Children’s Hospital, San Diego, CA 92123, USA; 2 Department of Pediatrics, School of Medicine, University of California San Diego, San Diego, CA 92093, USA; 3 Children’s Hospital Los Angeles, Los Angeles, CA 90027, USA; 4 Keck School of Medicine, University of Southern California, Los Angeles, CA 90033, USA; 5 Akron Children’s Hospital, Akron, OH 44302, USA; 6 Atrium Health Levine Children’s Hospital, Charlotte, NC 77030, USA; 7 Department of Pediatrics, School of Medicine, Wake Forest University, Winston-Salem, NC 27101, USA; 8 Penn State Children’s Hospital, Hershey, PA 17033, USA; 9 Cincinnati Children’s Hospital Medical Center, Cincinnati, OH 45229, USA; 10 Social Work Department, University of Wisconsin-River Falls, River Falls, WI 54022, USA; 11 Faculty of Writing and Literature, Lane Community College, Eugene, OR 97405, USA; 12 Division of Pediatric Rheumatology, Rutgers Robert Wood Johnson Medical School, New Brunswick, NJ 08901, USA

**Keywords:** pediatric rheumatology, burnout, wellness, resilience, narrative medicine

## Abstract

**Highlights:**

**What are the main findings?**

- Pediatric rheumatology fellows have high levels of burnout as well as high levels of resilience.
- Pediatric rheumatology fellows with more support for learning experienced less burnout and better well-being.
- Following the narrative medicine intervention, participants experienced a shift toward feelings that work is meaningful, as well as a shift toward feelings of having time for personal and family life.

**What are the implications of the main findings?**

- Pediatric rheumatology fellowship programs should focus on offering structured wellness programs and resilience-building initiatives, as well as organized learning opportunities.
- Future studies should investigate the utility of narrative medicine intervention for reducing burnout among pediatric rheumatology fellows on a larger scale.

**Abstract:**

**Background/Objectives**: Recognizing workforce shortages and adverse impacts of trainee emotional exhaustion, we aimed to measure baseline levels of wellness, resilience, and burnout among pediatric rheumatology fellows as well as to understand the feasibility of implementing a narrative medicine intervention in this population. **Methods**: This study involved pre- and post-participation questionnaires for pediatric rheumatology fellows in the United States and Canada who participated in a narrative medicine intervention. Survey components included the Mini Z Burnout Survey, the Mayo Well-being Index and the Connor-Davidson Resilience Scale (CD-RISC), baseline demographics and self-reported burnout-related factors. **Results**: All sixteen invited fellows completed the pre-participation survey, and 15 of the 16 participants completed the narrative intervention. The median (range) baseline CD-RISC resilience score was 73.5 (44–88), and the baseline Mini-Z Burnout Survey showed high levels of burnout with a median (range) of 34 (17–44). The median (range) score of the baseline Mayo Well-being Index was 3 (0–7), with scores of ≥4 associated with physicians at risk of suicidal ideation and/or serious thoughts of leaving medicine. Following participation in the narrative medicine intervention, surveys showed a shift toward feelings that work is meaningful, as well as a shift toward feelings of having time for personal and family life. **Conclusions**: In this pilot study, baseline levels of wellness, resilience, and burnout among pediatric rheumatology fellows highlighted target areas to mitigate burnout and maximize resilience among fellows. Narrative medicine is a feasible and acceptable intervention, and future larger studies should investigate the possible impact of narrative medicine on burnout among pediatric rheumatology fellows.

## 1. Introduction

Physician burnout is not new. Burnout, encompassing emotional exhaustion, depersonalization, and a lack of sense of accomplishment, has adversely impacted the delivery of high-quality, compassionate care across healthcare settings for years. Even prior to the pandemic, electronic medical records, performance metrics, billing requirements, and increased patient volume had drained the meaning out of medicine for many [1,2,3]. Across the medical realm, burnout has resulted in critical individual and organizational costs, with consequences such as fatigue, depression, suicidal ideation, suicide, anxiety, sleep disturbance, irritability, and substance abuse, as well as poor patient and provider satisfaction, missed work days, diminished job performance, and high turnover [3,4]. On the other hand, physicians have higher rates of resilience compared to the general population. However, while higher resilience scores have been associated with lower burnout rates, even the most resilient physicians have been found to have substantial burnout rates [5].

As is the case with other specialists, surveyed rheumatologists exemplify symptoms of burnout with high rates of emotional exhaustion and depersonalization and low rates of personal accomplishment [6]. This baseline burnout existed prior to the pandemic, and studies have shown that the pandemic further disrupted healthcare delivery and education among rheumatologists [7,8]. These studies, however, focus on pediatric rheumatology physicians who have completed training and do not investigate the underlying burnout rates among pediatric rheumatology trainees. The limited investigation of burnout among fellows within rheumatology has been focused on adult rheumatology fellows, with data collected pre-pandemic [9]. To date, there are limited data specifically investigating burnout, wellness, and resilience among pediatric rheumatology fellows, including understanding potential contributing factors to burnout for these trainees. These data are critical, particularly in a world of rising healthcare shortages and fewer trainees choosing to enter pediatric rheumatology.

It is important to identify the theoretical foundations when investigating relevant variables impacting burnout among pediatric rheumatology fellows. Shanafelt’s conceptual model of the drivers of burnout and engagement offers a framework of understanding, emphasizing the intersection between physician well-being and organizational contribution to burnout [10,11]. The model includes physician and organizational factors such as workload and job demands, control and flexibility, work–life integration, social support and community at work, organizational culture and values, efficiency and resources, and meaning in work. Research in the adult rheumatology literature reflects these contributors to burnout, where providers with higher burnout demonstrate higher rates of dissatisfaction with their specialty, low income, long work hours, lack of exercise, solo practice, and low personal accomplishment scores [6,12]. Studies of adult rheumatology fellows have additionally found burnout to be related to pager and patient demands, documentation, and presentations and expectations [9]. These elements offer insight into relevant variables that may contribute to burnout among pediatric rheumatology fellows.

In the context of rising levels of burnout in the medical community, it is critical to identify easily accessible, feasible interventions that can mitigate burnout. Narrative medicine has emerged as a protective and useful tool to improve wellness among trainees and physicians, offering a space for peer connection and emotional debriefing from stressful medical encounters [13]. Narrative medicine offers the opportunity to apply story to medical education and practice, building upon the Accreditation Council for Graduate Medical Education core competencies to develop professional and communication skills [14,15]. The creation of a peer-oriented reflective space is thought to minimize burnout and support resilience factors by supporting human connection. This model is supported by a study among palliative care fellows, where narrative medicine fostered resilience, community building, and reflective practice [16]. The current study is the first of its kind aimed at measuring baseline levels of wellness, resilience and burnout among pediatric rheumatology fellows based on Shanafelt’s conceptual model, and to monitor the feasibility of a narrative medicine intervention in this population.

### Research Hypotheses

Based on the literature reviewed on adult rheumatologists and rheumatology fellows, it was hypothesized that pediatric rheumatology fellows would have high rates of resilience as well as high levels of burnout. First-year fellows were expected to have the highest rates of burnout given increased clinical time requirements during the first year of fellowship. The narrative medicine intervention was anticipated to be a feasible intervention among pediatric rheumatology fellows, with an expected seventy percent of participants attending at least fifty percent of the narrative sessions.

## 2. Materials and Methods

This study involved pre- and post-participation surveys of pediatric rheumatology fellows in training programs within the United States and Canada who were recruited for a narrative medicine intervention led by two trained narrative medicine facilitators. Pre- and post-participation surveys included scales assessing burnout, wellness, and resilience, as well as baseline demographics. The narrative medicine intervention took place from March to April 2024 with four 1 h video-based sessions, and baseline survey data were collected within one to two months prior to the narrative medicine intervention, halfway through the training year. Supplementary Materials File S2 demonstrates the narrative medicine curriculum implemented.

### 2.1. Pre- and Post-Participation Surveys

The pre- and post-participation surveys included the Mini Z Burnout Survey, a 10-item scale with 5-point Likert scales and one open-ended question assessing burnout, stress, and satisfaction, which takes approximately 1–2 min to complete [17,18]. The Mini Z Burnout Survey has a Cronbach’s alpha of 0.8, showing good internal consistency and construct validity with drivers correlated at 0.26–0.46 [19]. Participants also completed the Connor-Davidson Resilience Scale (CD-RISC), a 25-item scale with 5-point Likert scales to address resilience, which takes approximately 5–7 min to complete [20,21]. The CD-RISC has good reliability with a Cronbach’s alpha of greater than 0.8 [22]. Studies show moderate to large correlations between the CD-RISC and measures of resilience, self-esteem, depression, and anxiety, supporting good convergent validity [23]. The Mayo Well-being Index, a 9-item scale measuring well-being and distress that takes 1–3 min to complete, was also included [24,25]. The Mayo Well-being Index has a Cronbach’s alpha of 0.68, showing moderate reliability, with a Content Validity Index of 0.94 and 0.91 for relevance and representativeness [25]. The Mini Z Burnout Survey, CD-RISC, and Mayo Well-being Index have all been used among a wide array of English-speaking healthcare professionals, including trainees previously, demonstrating a good fit for the current study sample. An additional questionnaire that focused on potential contributing factors to burnout was included. This questionnaire was informed by Shanafelt’s model referenced below, and it included items on demographic data (Supplementary Materials File S1). Areas of interest for burnout-related factors include time on call, outpatient clinic, joint injections, workplace culture and environment, colleagues and supervisors, salary, career growth, development and research, commuting, and sleep. This separate burnout-related questionnaire took approximately 5–7 min to complete and was only administered as part of the pre-participation survey. The burnout-related questionnaire was piloted among pediatric rheumatologists who had already completed fellowship training prior to implementation, and the questionnaire was adjusted based on pilot participant feedback.

### 2.2. Conceptual Model

The primary guiding conceptual model for this work is Shanafelt’s model of the drivers of burnout and engagement, which incorporates examples of individual, work unit, organizational, and national factors within specific driver dimensions [10,11]. Driver dimensions identified include workload and job demands, control and flexibility, work–life integration, social support and community at work, organizational culture and values, efficiency and resources, and meaning in work. For each driver, more optimal conditions lead to engagement, while less optimal conditions result in burnout. This simple yet comprehensive model demonstrates the intersection between physician well-being and the health of the organization, recognizing the importance of support from stakeholders at the organizational and national levels to mitigate burnout. The model offers an opportunity to target a specific dimension that may benefit from change for future interventions.

### 2.3. Population, Sampling, and Data Collection

Pediatric rheumatology fellows were the population of interest, with convenience sampling conducted to select a sample of fellows to participate in the study. Participants included fellows from small and large training programs at different levels of training, with a broad regional representation. Fellows from the United States included those in 3-year core pediatric rheumatology fellowships as well as those in combined fellowship training programs (e.g., rheumatology and infectious disease), and the fellow from Canada was in a 2-year core pediatric rheumatology fellowship. Participants completed the initial questionnaire, then participated in a narrative medicine intervention, and then completed a post-participation questionnaire.

Inclusion criteria included pediatric rheumatology fellows currently in training within the United States and Canada. Individuals who had graduated from their training in pediatric rheumatology fellowship were excluded from the study. At the time of the study, there were an estimated 109 filled pediatric rheumatology fellowship positions (including all training years) in the United States [26].

Data were collected through self-reported demographic data and participant-completed questionnaires through the University of Washington REDCap [27].

### 2.4. Statistical Analysis

Descriptive analyses were used for participant demographics. Using the RStudio 2023.09.1 (Build 494) Desert Sunflower Release for Windows, initial descriptive analyses were performed on all variables. Individual instrument scores were examined for ceiling and floor effects. All variables were examined to ascertain the data distributions and assessed for normality. Wilcoxon signed-rank tests were used, given that the data were not normally distributed. Effect size was not included in the analysis due to the small sample size.

## 3. Results

Of the 16 physicians who received an invitation to participate electronically, 16 (100%) completed the pre-participation survey, with demographics depicted in Table 1. Ninety-four percent of participants covered rheumatology-only services, while the remaining six percent covered both rheumatology and immunology at their hospital. Fifty-six percent of participants managed a primary rheumatology service in addition to consultations, and 44 percent of participants shared call with a co-fellow. The Shapiro–Wilk Test demonstrated that the data were not normally distributed, so Wilcoxon signed-rank tests were used as shown below.

The median (range) resilience score among 16 physicians who completed the pre-participation CD-RISC was 73.5 (44, 88) (Table 1), with high resilience defined as scores over 25.5 [20]. Notably, while the CD-RISC showed high rates of resilience, the Mini-Z Burnout Survey showed high levels of burnout with a median total score of 34 (range 17, 44), comprising a median subscale 1 score of 17 (range 8, 21) and a median subscale 2 score of 19 (range 9, 23). A total score on the Mini-Z Burnout Survey of 40 or higher suggests a joyful workplace. A score equal to or above 20 on the first subscale suggests a highly supportive practice, and a score equal to or above 20 on the second subscale suggests the office has a reasonable pace with manageable electronic medical record stress. Notably, when burnout scores were broken down by fellowship year, first-year fellows were noted to have higher levels of burnout, with total median (range) scores of 30 (24, 44) compared to 39 (34, 42) for second-year fellows and 33.5 (17, 39) for third-year fellows. Given that the data were non-parametric, Spearman’s correlation was calculated, which showed that the correlation between resilience and burnout score was statistically significant, where a higher level of resilience correlated with lower burnout scores (r = 0.5, *p*-value = 0.03). Spearman’s correlation between wellness scores and burnout scores similarly revealed a statistically significant correlation, where worse wellness scores correlated with worse burnout scores (r = −0.765, *p* = 0.006). Lastly, Spearman’s correlation between resilience and wellness scores showed a statistically significant correlation, where better wellness scores correlated with higher levels of resilience (r = −0.563, *p* = 0.023).

The median (range) score of the Mayo Well-being Index for 16 physicians who completed the pre-participation survey was 3 (0, 7), with scores of greater than or equal to 4 associated with physicians at risk of adverse consequences such as suicidal ideation and/or serious thoughts of leaving medicine. Thirty-eight percent of participants were noted to be at risk of adverse consequences based on Mayo Well-being Index scores. Notable highlights of potential burnout factors included poor sleep with a median (range) of 7 (5.5, 7) hours of sleep per night. The survey showed a mean (range) of 7 (5.5, 7) hours of sleep among first-year fellows, 6.5 (6, 7) hours of sleep among second-year fellows, and 6.5 (6, 7) hours of sleep among third-year fellows. Sleep did not correlate with well-being, resilience, or burnout scores when analyzed with Spearman’s correlation. Only twelve percent reported feeling wide awake with a clear head, with the remaining alertness ratings depicted in Table 2. Twenty percent of participants with dependents endorsed the availability of on-site daycare or other childcare benefits. Sixty-nine percent of participants reported feeling that they had no control over the scheduling of their clinics, and two-thirds of participants felt their workplace had a good culture. Three-quarters of participants felt leadership communicated well with team members, and sixty-three percent felt they were able to express concerns and frustrations to leadership. Eighty-one percent felt supported by colleagues and supervisors, and eighty-eight percent liked their colleagues and supervisors. Table 3 shows participant-reported ratings of supervisory gratitude for participant work, program support for career growth, and program support for learning. Spearman’s correlation revealed a statistically significant correlation between support for career growth and burnout (r = 0.582, *p*-value 0.0180), where those with better support for career growth experienced less burnout. A statistically significant correlation was also noted between support for learning and burnout (r = 0.647, *p* = 0.00672) as well as between support for learning and well-being (r = −0.552, p = 0.0265), demonstrating that those with more support for learning experienced less burnout and better well-being. Twelve percent of survey respondents were required by their program to apply for funding to support their salary. Sixty-nine percent of participants had a commute to work under 30 min, while the remainder had a commute time between 30 and 60 min. Thirty-one percent of participants traveled to a satellite clinic, with satellite commute timing ranging from 10 min to 70 min. Table 4 shows differences in weeks on call, clinics per week, and joint injections on call and off call.

**Figure 1:**
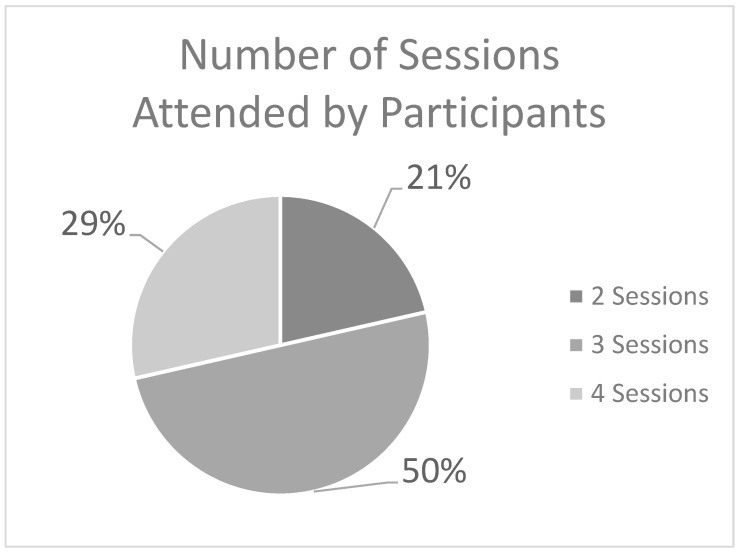
Number of sessions attended by participants.

**Figure 2:**
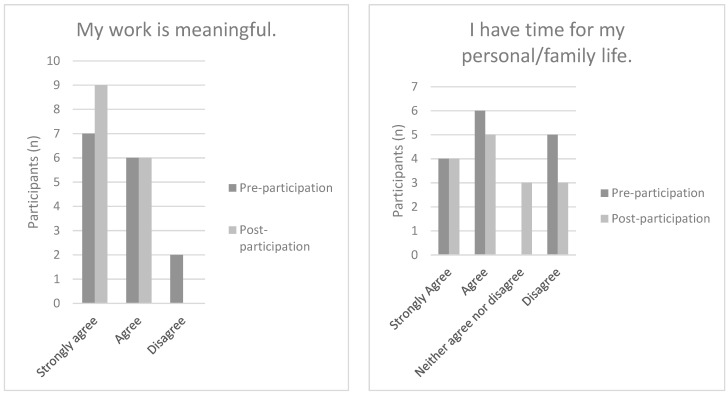
Pre- and post-participant responses reflecting on the meaning of work and time for family and personal life.

Fifteen of the sixteen initially consented participants completed two or more of the four narrative medicine sessions (Figure 1). The Wilcoxon signed-rank test revealed no statistically significant difference between pre-participation and post-participation Mayo Well-being (pre-median (range) = 3 (0–7), post-median (range) = 2 (0–6), *p* = 0.173), CD-RISC (pre-median (range) = 76 (44–88), post-median (range) = 75 (46–92), *p* = 0.495), or Mini-Z Burnout total scores (pre-median (range) = 34 (17–44), post-median (range) = 36 (30–43), *p* = 0.360). Mayo Well-being responses revealed a shift toward feelings that work is meaningful as well as a shift towards feelings of having time for personal/family life post-participation (Figure 2). Fifty-three percent rated the sessions as ‘Excellent,’ and forty-seven percent rated the sessions as ‘Very Good’. All but one participant were somewhat to very likely to recommend the intervention to a colleague. Seventy-nine percent felt the session length of one hour was ‘Just Right’, with fourteen percent suggesting the sessions were ‘A Bit Long’ and seven percent suggesting the sessions were ‘A Bit Short’. Following participation, sixty percent of participants were interested in sharing their writings with others in a public format. Participant quotes regarding the impact of the intervention are listed in Table 5 to illustrate participants’ experiences.

## 4. Discussion

### 4.1. Theoretical Implications

The high levels of resilience among physicians found in this study are not unexpected, as several studies have shown that physicians exhibit higher levels of resilience than the general working population [5,28]. Importantly, however, prior studies have shown resilience to be inversely associated with burnout, suggesting an interesting avenue for potential intervention moving forward [5]. This study reinforces previously established patterns in other fields, demonstrating that individuals with high levels of resilience have lower rates of burnout. It also highlights the urgency of addressing the ongoing physician shortages in pediatric rheumatology, recognizing that burnout among fellows may deter future trainees from joining the field [9].

Future studies should investigate the potential influence of training programs with more than one fellow per year compared to programs with only one fellow total or one fellow per year. It may be of interest for future studies to identify an association between generational assignment and resilience, comparing Generation X, millennials, and Zoomers, recognizing a potential difference in cultural attitudes and understanding of burnout and resilience.

### 4.2. Practical Implications

When looking at potential factors of interest that contribute to burnout among fellows, there were several areas that may serve as points of interest for future intervention. The data, for example, demonstrated that twenty percent of participants with dependents endorsed the availability of on-site daycare or other childcare benefits, suggesting room for improvement in support for those with families. Other areas that may benefit from future intervention include removing the requirement for fellows to apply to support their own funding and shifting that burden to administration, recognizing that the non-clinical burden is shown in this study to correlate with elevated rates of burnout. Data from this study demonstrated that those with more support for learning experienced less burnout and better well-being, suggesting programs should focus on offering structured wellness programs and resilience building, as well as organized learning opportunities. These areas of interest align with Shanafelt’s conceptual model, acknowledging the relevance of institutional and organizational factors when considering provider burnout.

A recent national study of burnout among rheumatology fellows in the United States found worse levels of fatigue, quality of life, and burnout to be associated with early trainee levels and younger age [9]. Thematic analysis of open-ended responses identified potentially mitigating factors for burnout, including exercise, family and friends, sleep, support at work, and hobbies. Respondents identified contributing factors to burnout, including pager, documentation, long hours, patient care demands, presentations, and expectations. Notably, both McGoldrick et al. and the current authors demonstrate worse burnout among first-year fellows. Unlike McGoldrick et al.’s study, the current study includes a broader investigation of potential burnout-associated factors, demonstrating that better support for career growth and support for learning are associated with less burnout. The current study also highlights potential areas for improvement, including access to daycare and requirements to apply for grant funding.

### 4.3. Understanding the Narrative Medicine Impact

Limited studies have investigated the potential feasibility and acceptability of a narrative medicine intervention for pediatric rheumatology trainees. Participant attendance rates and responses to the narrative medicine intervention in this study demonstrate the feasibility and acceptability of narrative medicine among pediatric rheumatology fellows. As this is a pilot feasibility study, it is not surprising that there were no statistically significant differences in pre- and post-participation measures of well-being or burnout. The shift toward feeling that work was meaningful, as well as a shift toward feelings of having time for personal and family life post-participation, suggests future larger studies should investigate the potential utility of narrative medicine to address burnout-related factors.

### 4.4. Limitations

There was significant diversity in program organization by geographic location and fellowship year, demonstrating heterogeneous program structures across the country. One limitation of this study is that it is difficult to account for other areas of diversity across programs that may influence burnout. Importantly, the sampled population includes fellows who had no call as well as those with up to 42 weeks of call per year, suggesting potential major influences on data analysis in this small sample size. Future studies should focus on examining the key factors contributing to the success of programs with low burnout rates. By understanding what sets these programs apart, educators and administrators can develop targeted strategies to revamp programs with high burnout rates, ultimately promoting a healthier and more sustainable learning environment.

This study is at risk of selection bias, as participant data were collected through convenience sampling in the course of a larger study integrating a narrative medicine intervention for participants. The small sample size emphasizes the need for larger studies to be appropriately powered in order to best understand the impact of narrative medicine on pediatric rheumatology fellow burnout. While only 16 participants were included, recognizing that there are typically only 20 to 30 pediatric rheumatology fellows trained in the United States annually, with typically three years total in training, this is not an insignificant representation of current fellows. Future studies should assess burnout and resilience levels by incorporating all pediatric rheumatology fellows rather than focusing only on pre-selected narrative medicine participants. Future studies should also consider including a control group for the narrative medicine intervention to better assess the intervention’s impact. Inherent in the study design is the risk of common method bias, recognizing that all variables of interest rely on self-reported measures. As this bias can affect the reliability and validity of the results, future studies should consider randomizing clear, specific, and unambiguous questions, which can mitigate common method bias.

There was a potential for sample bias in baseline burnout-related data, recognizing that participants were invited to complete the study as part of a larger narrative medicine intervention. Another limitation of the study is that the survey structure did not incorporate information regarding participant mental health-related concerns, including potential underlying psychiatric illnesses and/or psychological supports in place. Recognizing the stigma around mental health in the medical field and the risk of item non-response, this topic of interest was omitted from the current study. Future studies, however, should consider targeting this specific area of interest to understand potential mitigating and exacerbating factors for burnout among pediatric rheumatology fellows.

## 5. Conclusions

This pilot study is the first of its kind, providing a snapshot of wellness, resilience, and burnout levels among pediatric rheumatology fellows with an associated feasible and acceptable narrative medicine intervention, contributing novel insights to the existing literature and addressing a critical knowledge gap. Future studies should consider the implementation of a narrative medicine intervention on a larger scale for pediatric rheumatology fellows.

## Figures and Tables

**Table 1:** Demographic characteristics of participants with numbers and percentages.

Demographic Characteristics	N (%)
Gender	
Male	3 (19)
Female	13 (81)
Age Range (years)	
25–30	4 (25)
31–35	7 (44)
36–40	4 (25)
41–45	1 (6)
Self-Identified Race	
Asian	3 (19)
Black or African American	1 (6)
Other	1 (6)
White	11 (69)
Self-Identified Ethnicity	
Hispanic	3 (19)
Non-Hispanic	13 (81)
Marital Status	
Single	6 (38)
Married	9 (56)
Divorced	1 (6)
Children or Dependents	5 (31)
Institution Geographic Location	
Pacific West U.S. ^1^	3 (19)
Mountain West U.S.	2 (12)
Midwestern U.S.	4 (25)
Southeastern U.S.	2 (12)
Northeastern U.S.	4 (25)
Canada	1 (6)
Year in Fellowship Training	
1	7 (44)
2	4 (25)
3	4 (25)
4	1 (6)

^1^ U.S. = United States.

**Table 2:** Sleepiness as rated on the Alertness Scale.

Alertness	Participants N (%)
Wide awake, fully awake, functioning at a high level; head clear	2 (12)
Functioning at a high level, not at peak, able to concentrate	6 (38)
Relaxed; awake; not at full alertness; responsive	2 (12)
A little groggy; clearly not at peak; let down	3 (19)
Fogginess; beginning to lose interest in remaining awake; slowed down	3 (19)

**Table 3:** Participant-reported rating of supervisory gratitude for participant work, program support for fellow career growth, and program support for fellow learning.

Participant-Reported Rating	Poor N (%)	Marginal N (%)	Satisfactory N (%)	Good N (%)	Optimal N (%)
Supervisory gratitude for participant work	3 (19)	2 (12)	2 (12)	8 (50)	1 (6)
Program support for fellow growth	1 (6)	3 (19)	2 (12)	6 (38)	4 (25)
Program support for fellow learning	0 (0)	3 (19)	4 (25)	4 (25)	5 (31)

**Table 4:** Differences in weeks on call, clinics per week, and joint injections on call and off call. Fellowship year four was omitted due to small numbers. Responses include participant-reported estimates.

Item Measured	Participant Responses
	**Median (Range)**
*Weeks on Call*	12 (0,42)
*Half-Day Clinics Per Week On Call*	**Number Clinics:** **N (%)**
0	0 (0)
1	7 (44)
2	4 (25)
3	2 (12)
4	1 (6)
5	1 (6)
8	1 (6)
*Half-Day Clinics Per Week Off Call*	
0	1 (6)
1	4 (25)
2	4 (25)
3	2 (12)
4	2 (12)
5	2 (12)
8	1 (6)
*Average Joint Injections Per Week On Call*	**Number Injections:** **N (%)**
0	10 (63)
0.1	1 (6)
0.5	1 (6)
1	2 (12)
2	1 (6)
3	1 (6)
*Average Joint Injections Per Week Off Call*	
0	12 (75)
0.1	1 (6)
0.5	0 (0)
1	3 (19)
2	0 (0)
3	0 (0)

**Table 5:** Participant responses describing the impact of participation on personal perspectives.

Participant	Response
1	The sessions helped remind me to look for the beauty and wonder of what we do every day. I enjoyed connecting with other fellows in this way. Reflecting on our shared experiences was nice, especially since I am finishing [my] fellowship this year.
2	It taught me to talk about [what I] feel without fear and overthinking.
3	I have to be honest. I think this would have been much more helpful during my first year and during residency, when I was much [busier] clinically. For now, I have a really good and flexible schedule and am at a really supportive institution. My work–life balance was great going into these sessions. What HAS improved is my feeling of love and care for my fellow peds rheum colleagues, and I look forward to seeing my group at future conferences.
4	I think a combination of the sessions and the fact that [we are] approaching spring and coming out of seasonal depression time [was] simultaneously positive for me. I definitely feel less burned out than when I started, though that is likely multifactorial. I think the most helpful part of the sessions in shifting my mood was just being encouraged to stop for an hour and think through some of the mess of emotions from the day/week and putting a pen to paper and putting words to those feelings so I could process and let some of the negativity go.
5	I think they have encouraged me to reflect more on my experiences with patients and coworkers and look at these experiences from many different lenses.
6	I have learned to appreciate art and try to delve into the meaning of each painting.
7	The ambiguity session reminded me that uncertainty and ambiguity can be a strength. That came at a perfect time in fellowship, where I was starting to drown a bit in seeing ambiguity and uncertainty as a failure. That lesson has stuck with me. I also have felt less alone in my own struggles with ambiguity/uncertainty and wounded healer. It has been healing to know that other fellows are feeling similarly and that we can get through this together.
8	[It has] helped me remember to look at the whole picture rather than the immediate problem in front of me.
9	I enjoyed the time for self-reflection and the opportunity to meet other fellows.
10	Yes, made me appreciate art more.
11	Hearing other fellows’ perspectives about fellowship and their trajectories was great. The sessions were led as safe spaces, and everyone was able to share their writings and [points of view], which was very meaningful and touching, too.
12	I think I am more self-reflective and try to have time in my day to self-reflect on my job.
13	Hard to tell with just a few sessions, but they kind [of] boosted my morale.

## Data Availability

Data is available upon reasonable request of the authors.

## Supplementary Materials

The following supporting information can be downloaded at: https://www.mdpi.com/article/10.3390/healthcare14132025/s1, File S1: Pediatric Rheumatology Fellow Questionnaire. File S2: Curriculum Outline. Refs. [29,30,31] are cited in Supplementary Materials.

## Abbreviations

The following abbreviations are used in this manuscript:

CD-RISC	Connor-Davidson Resilience Scale
