# Supplementary material for: Wellness, Resilience, and Burnout Among Pediatric Rheumatology Fellows: A Narrative Medicine Pilot Intervention
Source: Healthcare (Basel). 2026 Jul 7;14(13):2025. doi: 10.3390/healthcare14132025 (PMC13362230; doi:10.3390/healthcare14132025)
Supplement: Supplementary file 1 [file healthcare-14-02025-s001.zip › File S1. Pediatric Rheumatology Fellow Questionnaire.pdf]

PEDIATRIC RHEUMATOLOGY FELLOW QUESTIONNAIRE

STUDY ID: ________________

Directions

In this booklet, you will find questions that ask you to consider your current overall wellbeing, resilience and burnout, as well as assess some potential contributing factors for burnout. We will also ask you to provide some basic information about yourself.

Please answer the questions on the questionnaire as best as you can. It will take you approximately 45 minutes to 1 hour to complete.

Your participation is completely voluntary. You may decline or leave any questions blank you do not wish to answer.

Please remember to hit the submit button following completion of each survey.

WELLBEING

In this section, we ask you to consider your level of wellbeing.

For Q 1-7, please circle ONE answer to indicate the extent to which you agree or disagree with each statement. Please consider your responses over the past month.

1. During the past month, have you felt burned out from your work?	**Yes**	**No**
2. During the past month, have you worried that work is hardening you emotionally?	**Yes**	**No**
3. During the past month, have you often been bothered by feeling down, depressed or hopeless?	**Yes**	**No**
4. During the past month, have you fallen asleep while sitting inactive in a public place?	**Yes**	**No**
5. During the past month, have you felt that all things you had to do were piling up so high that you could not overcome them?	**Yes**	**No**
6. During the past month, have you been bothered by emotional problems (such as feeling anxious, depressed, or irritable)?	**Yes**	**No**
7. During the past month, has your physical health interfered with your ability to do your daily work at home and/or away from home?	**Yes**	**No**

For Q 8-9, please rate your level of agreement with each statement.

8. The work I do is meaningful to me.	**1-Strongly Agree**	**2-Agree**	**3-Neither agree nor Disagree**	**4-Disagree**	**5-Strongly Disagree**
9. My work schedule leaves me enough time for my personal/family life.	**1-Strongly Agree**	**2-Agree**	**3-Neither agree nor Disagree**	**4-Disagree**	**5-Strongly Disagree**

RESILIENCE

In this section, we ask you to consider your level of resilience.

For Q 1-25, please circle ONE answer to indicate the extent to which you agree or disagree with each statement. Please consider your responses over the past month.

1. I am able to adapt when changes occur.	**0-Not true At All**	**1-Rarely True**	**2-Sometimes True**	**3-Often True**	**4-True nearly All the Time**
2. I have one close and secure relationship.	**0-Not true At All**	**1-Rarely True**	**2-Sometimes True**	**3-Often True**	**4-True nearly All the Time**
3. Sometimes fate or God helps me.	**0-Not true At All**	**1-Rarely True**	**2-Sometimes True**	**3-Often True**	**4-True nearly All the Time**
4. I can deal with whatever comes my way.	**0-Not true At All**	**1-Rarely True**	**2-Sometimes True**	**3-Often True**	**4-True nearly All the Time**
5. Past successes give me confi-dence.	**0-Not true At All**	**1-Rarely True**	**2-Sometimes True**	**3-Often True**	**4-True nearly All the Time**
6. I try to see the humorous side of things when I am faced with problems.	**0-Not true At All**	**1-Rarely True**	**2-Sometimes True**	**3-Often True**	**4-True nearly All the Time**
7. Having to cope with stress can make me stronger.	**0-Not true At All**	**1-Rarely True**	**2-Sometimes True**	**3-Often True**	**4-True nearly All the Time**
8. I tend to bounce back after illness, injury or other hardships.	**0-Not true At All**	**1-Rarely True**	**2-Sometimes True**	**3-Often True**	**4-True nearly All the Time**
9. I believe most things happen for a reason.	**0-Not true At All**	**1-Rarely True**	**2-Sometimes True**	**3-Often True**	**4-True nearly All the Time**
10. I make my best effort, no matter what.	**0-Not true At All**	**1-Rarely True**	**2-Sometimes True**	**3-Often True**	**4-True nearly All the Time**

RESILIENCE-CONTINUED

11. I believe I can achieve my goals, even if there are obstacles.	**0-Not true At All**	**1-Rarely True**	**2-Sometimes True**	**3-Often True**	**4-True nearly All the Time**
12. I believe I can achieve my goals, even if there are obstacles.	**0-Not true At All**	**1-Rarely True**	**2-Sometimes True**	**3-Often True**	**4-True nearly All the Time**
13. In times of stress, I know where to find help.	**0-Not true At All**	**1-Rarely True**	**2-Sometimes True**	**3-Often True**	**4-True nearly All the Time**
14. Under pressure, I stay focused and think clearly.	**0-Not true At All**	**1-Rarely True**	**2-Sometimes True**	**3-Often True**	**4-True nearly All the Time**
15. I prefer to take the lead in problem-solving.	**0-Not true At All**	**1-Rarely True**	**2-Sometimes True**	**3-Often True**	**4-True nearly All the Time**
16. I am not easily discouraged by failure.	**0-Not true At All**	**1-Rarely True**	**2-Sometimes True**	**3-Often True**	**4-True nearly All the Time**
17. I think of myself as a strong person when dealing with life's challenges and difficulties.	**0-Not true At All**	**1-Rarely True**	**2-Sometimes True**	**3-Often True**	**4-True nearly All the Time**
18. I make unpopular or difficult decisions.	**0-Not true At All**	**1-Rarely True**	**2-Sometimes True**	**3-Often True**	**4-True nearly All the Time**
19. I am able to handle unpleas-ant or painful feelings like sad-ness, fear, and anger.	**0-Not true At All**	**1-Rarely True**	**2-Sometimes True**	**3-Often True**	**4-True nearly All the Time**
20. I have to act on a hunch.	**0-Not true At All**	**1-Rarely True**	**2-Sometimes True**	**3-Often True**	**4-True nearly All the Time**
21. I have a strong sense of pur-pose in life.	**0-Not true At All**	**1-Rarely True**	**2-Sometimes True**	**3-Often True**	**4-True nearly All the Time**
22. I feel like I am in control.	**0-Not true At All**	**1-Rarely True**	**2-Sometimes True**	**3-Often True**	**4-True nearly All the Time**

RESILIENCE-CONTINUED

23. I like challenges.	**0-Not true At All**	**1-Rarely True**	**2-Sometimes True**	**3-Often True**	**4-True nearly All the Time**
24. I work to attain goals.	**0-Not true At All**	**1-Rarely True**	**2-Sometimes True**	**3-Often True**	**4-True nearly All the Time**
25. I take pride in my achievements.	**0-Not true At All**	**1-Rarely True**	**2-Sometimes True**	**3-Often True**	**4-True nearly All the Time**

BURNOUT

In this section, we ask you to consider your level of burnout.

For Q 1-10, please indicate the best answer.

1. Overall, I am satisfied with my current job.	**1-Strongly Agree**	**2-Agree**	**3-Neither agree nor Disagree**	**4-Disagree**	**5-Strongly Disagree**
2. I feel a great deal of stress because of my job.	**1-Strongly Agree**	**2-Agree**	**3-Neither agree nor Disagree**	**4-Disagree**	**5-Strongly Disagree**

3. Using your own definition of “burnout,” please circle one of the answers below:

a. I enjoy my work. I have no symptoms of burnout.

b. I am under stress, and don’t always have as much energy as I did, but I don’t feel burned out.

c. I am definitely burning out and have one or more symptoms of burnout, e.g. emotional exhaustion.

d. The symptoms of burnout that I’m experiencing won’t go away. I think about work frustrations a lot.

e. I feel completely burned out. I am at the point where I may need to seek help.

4. My control over my workload is:	**1-Poor**	**2-Marginal**	**3-Satisfactory**	**4-Good**	**5-Optimal**
5. Sufficiency of time for completing my work is:	**1-Poor**	**2-Marginal**	**3-Satisfactory**	**4-Good**	**5-Optimal**
6. Which number best describes the atmosphere in your primary work area?	**Calm 1**	**2**	**Busy, but reasonable 3**	**4**	**Hectic, Chaotic 5**

BURNOUT-CONTINUED

7. My professional values are well-aligned with those of my direct leaders	**1-Strongly Agree**	**2-Agree**	**3-Neither agree nor Disagree**	**4-Disagree**	**5-Strongly Disagree**
8. The degree to which my team works efficiently together is:	**1-Poor**	**2-Marginal**	**3-Satisfactory**	**4-Good**	**5-Optimal**
9. The amount of time I spend on work at home is:	**1-Excessive**	**2-Moderately High**	**3-Satisfactory**	**4-Modest**	**5-Minimal /None**
10. My work day is mainly frustrating:	**1-Strongly Agree**	**2-Agree**	**3-Neither agree nor Disagree**	**4-Disagree**	**5-Strongly Disagree**

11. Tell us more about your stresses and what we can do to minimize them:

____

____

____

____

BURNOUT FACTORS

In this section, we ask you to provide information on factors that may influence burnout.

For Q 1-23, please circle ONE answer that best reflects your response. Where there are blanks, please fill in the blank. Please consider your responses over the past month.

Time On Call

1. How many weeks out of 52 weeks of the year are you on call?

   a. _______ Weeks out of 52 weeks.

   b. Prefer not to answer

2. Do you share call load with a co-fellow?

   a. Yes

   b. No

   c. Prefer not to answer

3. How many weeks out of your call do you have another fellow on call at the same time as your call?

   a. _______ weeks out of 52 weeks

   b. Unsure

   c. None

   d. Prefer not to answer

4. Do you have a primary rheumatology service that you take care of?

   a. Yes

   b. No

   c. Other: ____

   d. Prefer not to answer

BURNOUT FACTORS-CONTINUED

Time On Call

5. What areas does your call service cover at your institution?

a. Rheumatology only

b. Rheumatology and Immunology

c. Other: ____

d. Prefer not to answer

Outpatient Setting

6. How many half-day clinics do you have on a typical on-call week?

a. _______ half-day clinics

b. Prefer not to answer

7. How many half-day clinics do you have on a typical off-call week?

a. _______ half-day clinics

b. Prefer not to answer

8. Do you feel you have control over your clinical schedule?

a. No—clinic schedule is dictated by administration without input from fellow for timing of clinic.

b. Somewhat—within pediatric rheumatology, a fellow can choose days of the week but not timing of schedule.

c. Yes—within pediatric rheumatology, clinic date and timing can be adjusted as long as number of patients seen is maintained.

d. Other: ____

e. Prefer not to answer

BURNOUT FACTORS-CONTINUED

Joint Injections

9. How many joint injections do you complete on a typical on-call week?

 a. ______ (#) joint injections

 b. Prefer not to answer

10. How many joint injections do you complete on a typical off-call week?

 a. ______ (#) joint injections

 b. Prefer not to answer

Workplace Culture & Environment

11. Do you feel your work place has a good culture?

 a. Yes

 b. No

 c. Other: ____

 d. Prefer not to answer

12. To what degree are you shown gratitude for hard work from your institution?

 a. Poor

 b. Marginal

 c. Satisfactory

 d. Good

 e. Optimal

 f. Prefer not to answer

BURNOUT FACTORS-CONTINUED

Workplace Culture & Environment

13. Do you feel leadership at your institution communicates plans, changes and expectations well?

a. Yes

b. No

c. Other: ____

d. Prefer not to answer

14. Do you feel you can bring concerns and frustrations to leadership and be heard?

a. Yes

b. No

c. Other: ____

d. Prefer not to answer

Colleagues and Supervisors

15. Do you like the immediate people you work with on a daily basis?

a. Yes

b. No

c. Other: ____

d. Prefer not to answer

16. Do you feel supported by the immediate people you work with on a daily basis?

a. Yes

b. No

c. Other: ____

d. Prefer not to answer

BURNOUT FACTORS-CONTINUED

Salary

17. Are you required to apply for funding to support your salary?

a. Yes

b. No

c. Other: ____

d. Prefer not to answer

Career Growth, Development and Research

18. To what degree do you feel your program offers support for personal career growth?

a. Poor

b. Marginal

c. Satisfactory

d. Good

e. Optimal

f. Prefer not to answer

19. To what degree do you feel your program offers support for learning?

a. Poor

b. Marginal

c. Satisfactory

d. Good

e. Optimal

f. Prefer not to answer

BURNOUT FACTORS-CONTINUED

Commuting

20. How long is your typical commute to work?

a. Less than 30 minutes

b. 30-60 minutes

c. >60 minutes

d. Prefer not to answer

21. Do you travel to satellite locations?

a. Yes

b. No

c. Other: ____

d. Prefer not to answer

If you answered YES to the previous question, please complete the following:

21a. How many satellite locations do you need to travel to?

a. ________________ (#) sites

b. Prefer not to answer

21b. How many days per week do you commute to the satellite locations?

a. ________________ days per week

b. Prefer not to answer

21c. What is the length of travel (in minutes) required on average to commute to the satellite locations?

a. _________________ minutes

b. Prefer not to answer.

BURNOUT FACTORS-CONTINUED

Sleep

22. How many hours of sleep per night did you have on average this past week?

  a. _____________ hours

  b. Prefer not to answer

23. Circle ONE response that best describes your level of alertness or sleepiness RIGHT NOW.

  a. Wide awake, fully awake, functioning at high level; head clear.

  b. Functioning at a high level, but not at peak; able to concentrate.

  c. Relaxed; awake; not at full alertness; responsive.

  d. A little groggy; clearly not at peak; let down.

  e. Fogginess; beginning to lose interest in remaining awake; slowed down.

  f. Sleepiness; prefer to be lying down; fighting sleep, woozy.

  g. Almost in reverie; sleep onset soon; lost struggle to remain awake.

DEMOGRAPHICS

In this section, we ask you to provide information on demographics

For Q 1-10, please circle ONE answer that best reflects your response. Where there are blanks, please fill in the blank. Please consider your responses over the past month.

1. What gender do you identify with?

- ☐ Male
- ☐ Female
- ☐ Non-binary
- ☐ Prefer not to answer

2. What age range do you fall within?

- ☐ 20-25
- ☐ 25-30
- ☐ 30-35
- ☐ 35-40
- ☐ >40
- ☐ Prefer not to answer

3. Marital Status:

- ☐ Single
- ☐ Married
- ☐ Domestic Partnership
- ☐ Prefer not to answer

4. Do you have children or other dependents (e.g. elderly parent)?

- ☐ Yes
- ☐ No
- ☐ Prefer not to answer

DEMOGRAPHICS

5. Does your institution/employer provide onsite daycare or other childcare benefits?

☐ Yes

☐ No

☐ Unsure

6. Institution geographic location?

☐ Northwestern U.S.

☐ Northeastern U.S.

☐ Southeastern U.S.

☐ Southwestern U.S.

☐ Midwestern U.S.

☐ Other: ____

☐ Prefer not to answer

7. In which state/province do you practice?

☐ ______________________________

☐ Prefer not to answer

8. How many rheumatology attendings (including APPs) are there within your division?

☐ 3 or less

☐ 3-5

☐ 6-10

☐ 10+

☐ Prefer not to answer

9. How many rheumatology fellows (including yourself) are there within your institution?

☐ ______________________ (#)

☐ Prefer not to answer

DEMOGRAPHICS

10. What year in fellowship are you?

☐ 1 ☐ 2 ☐ 3 ☐ 4 ☐ Other: _______________ ☐ Prefer not to answer

Thank you

Thank you for participating in our survey!

Please remember to hit the submit button following completion of each survey.

PEDIATRIC RHEUMATOLOGY FELLOW QUESTIONNAIRE

THANK YOU FOR YOUR PARTICIPATION
