# Supplementary material for: Wellness, Resilience, and Burnout Among Pediatric Rheumatology Fellows: A Narrative Medicine Pilot Intervention
Source: Healthcare (Basel). 2026 Jul 7;14(13):2025. doi: 10.3390/healthcare14132025 (PMC13362230; doi:10.3390/healthcare14132025)
Supplement: Supplementary file 1 [file healthcare-14-02025-s001.zip › File S2. Curriculum Outline.pdf]

Curriculum Outline

Session Format:

Please note that for facilitators who prefer a general bulleted outline of topics to discuss, please refer to the blue wording in this curriculum outline.

Session I:

Part I: Art and Medicine (30 min)

Introduction: (To be given by the Course Instructor; two minutes)

Welcome, everyone, to the first of four sessions that we will be spending together in the coming months. We hope you will enjoy these sessions and find some time and space to connect with each other. My name is ___, and I am pleased to introduce ______ who will be welcoming us into the space through an introductory talk on art and medicine. Today's session will focus on medicine reflected through art and how art can be used for restorative purposes. We will hear from ___ for the first half of our session, then spend some time writing. After writing, we will have time for introductions and expectations, and then we will have space to share our writings with one another, followed by a closing for the session. Please make sure to have a pen and paper or a computer available for use when the writing portion of our sessions comes around. If you have any questions throughout, please feel free to message me privately in the chat or send me a message or email after today's session. Without further ado, I will pass it on to ____.

Lecture (30 min) *Note this lecture is given by a separate provider, facilitators need not lead this portion. Below is the general outline of this portion of the lecture:*

The importance of observation cannot be underscored in medicine. Several institutions have employed art discussions and observation of artistic details as training mechanisms. Several art pieces depicting illness are also very useful for trainees to understand and appreciate the perception and depiction of the illness by the artist. Understanding that several people may have different points of view about a painting is important and can be applied to medical training. Burnout is unfortunately very prevalent among medical professionals. Staying open to the uncertainty in art and the myriad emotions evoked by artworks could help physicians through difficult encounters. Lastly, art can be used as a vehicle for wellness and relaxation. The objectives of this 30 min presentation are listed below.

Objectives:

1. *Discuss the importance of observation in physical diagnosis, and recognize some examples of medical conditions depicted in works of art.*
2. *Discuss how observation and discussion of works of art can help in understanding uncertainty and emotions evoked when providing medical care to patients.*
3. *Experience a wellness activity—a time of engagement and relaxation—while observing and discussing works of art.*

Writing Prompt: (Introduced in the larger group by the Course Instructor; eight minutes)

Thank you ___ for that wonderful introduction to the overlap of art and medicine. Sometimes, we look to art for medically related images as an outlet or expression of our daily experiences. As many of you know, in pediatric rheumatology, we often see and cope with difficult cases and sometimes devastating outcomes, and it can be hard to find an outlet to process these experiences. Sometimes, rather than reflect our day-to-day experiences, art can serve as an outlet. Inspiration from art can offer a form of tranquility and peace from these otherwise stressful scenarios. Today, we will be looking at some shared art to inspire that sense of peace.

Before we move to the art, I invite you to close your eyes and notice in your body any sensations, tightness, or breathing patterns. Notice if you feel yourself clenching your teeth, slumping your shoulders, or notice the feeling of your feet against the ground, or of your back against the chair. Focus on your breathing as you breathe in and out. And when you are ready, open your eyes. As you will see in the photos below, today we offer several photos of artistic inspiration to allow for a restorative space. You will see Francois Girard's 'Inspiration,' 'Surreal art' by Jim Warn, 'Wave of Life,' by Mary Tantillo, French's photorealistic painting, and Charis Tsevis' African Bricks mosaic. As you look at these images, choose one that speaks best to you. Using your five senses, write about what the image elicits for you. Do you feel the splash of the water coming off the canvas? Do you smell the scent of the ocean breeze? Do you feel the crevices between the mosaic tiles? Take five minutes to write, and we will notify you when you have one minute left. When we are done writing, we will invite everyone to come back together and share their experiences. *[When there is one minute left, the facilitator asks: What else is also true?]*

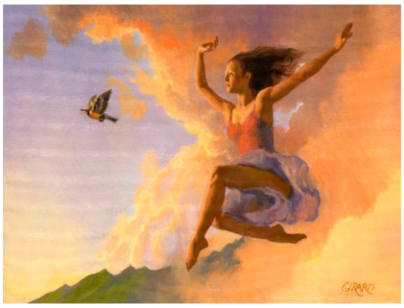

Inspiration, by Francois Girard

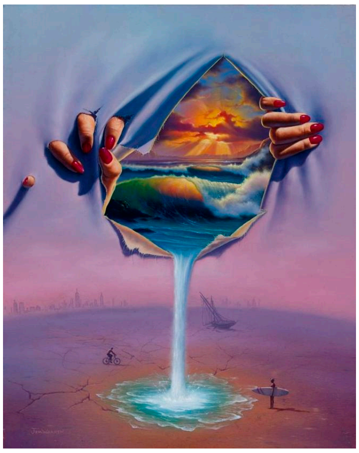

Surreal Art, by Jim Warren (https://myartmagazine.com/art-inspiration/daily-art-inspiration-1073)

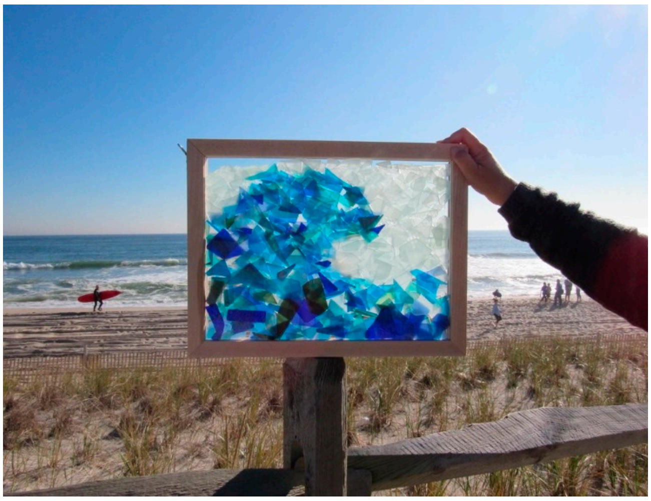

Wave of Life, by Mary Tantillo (https://www.swellcolors.com/reclaimed/sea-glass-inspired-seascapes-surfer-gift)

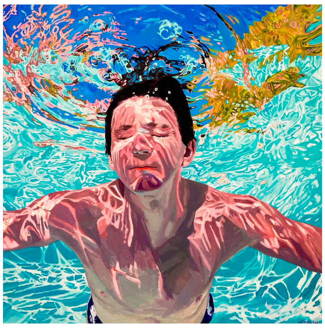

Bright Side: Photorealist Figurative Painting of Young Man in Aqua Blue Pool, by Samantha French

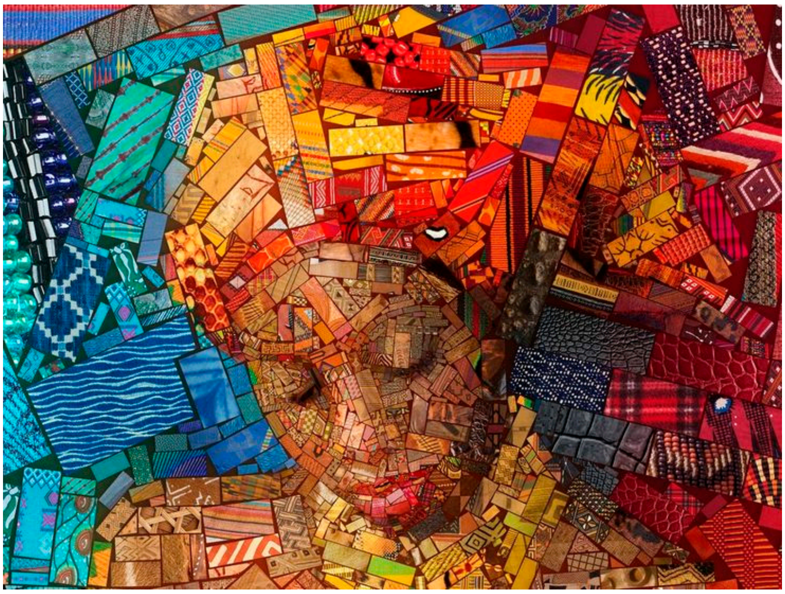

The African Bricks “The Pap Lady” by Charis Tsevis (https://tsevis.com/)

Part II: Break-Out Sessions (25 min)

Facilitators and group members will be sent to a separate break-out room to share

prompt writings with one another.

<u>Facilitator 1</u>: **Introductions** (five minutes)

Welcome to our small group. As we meet together in this shared space, we want to ensure everyone feels comfortable and safe to share with one another. Please remember that what’s shared in today’s session stays within today’s session, unless there is concern for anyone potentially harming themselves or others. We invite you all to introduce yourselves to each other, and please share your name, location, year of training, and favorite pastime activity.

[Below is a list of relevant points for facilitators who prefer not to follow the above script.

- *Welcome to the shared space*
- *What’s shared in the session stays in the session, unless there is concern for harming self or others*
- *Introductions: Name, location, year of training, favorite non-work activity]*

Set expectations (four minutes)

Now that we have all done brief introductions, it is time to set expectations for these sessions, so we all know what to expect during our session today. These are the areas we can start with, and if you have other areas you would like to incorporate as we go, please feel free to add them. We will save these as a reference for future sessions if anyone has concerns that arise and wants to refer back to our group expectations. *[Facilitator 2 will type expectations on the document to be saved and shared on screen with the group, to be shared at the beginning of each session]*

[Below is a list of relevant points for facilitators who prefer not to follow the above script.

- *Time to set expectations.*
- *List the items below to start with, open to the group to add others*
- *We will save these as a reference in case anyone has concerns]*
- Confidentiality, meaning what's said on this Zoom call stays on this Zoom call. As was mentioned to each of you during the consent process, if we are concerned that you or someone else may be harmed, it may need to be shared with appropriate individuals. Otherwise, please keep it in this room!
- Speak from the first person and talk from personal experience, using the terms "I—me—my"
- Generous listening, meaning no distractions and minimal interruptions if possible. If you are willing/able, put your phone on silent on the other side of the room.
- Allow for differences. There is no need to agree, but a need to hear each other and allow space for each person's opinions.
- Avoid giving unsolicited advice, commentary, or invasive probing; No teaching if at all possible.
- Start on time
- No eating
- Allow for silence.
- Cameras on during the sessions when not writing.

Facilitator 2: **Prompt Review:** (10 min)

Now it is time to share. Who would like to share a word, phrase, or what they wrote? What

did you see or hear? How did it feel? Did anything in what you heard remind you of anything?

[Below is a list of relevant points for facilitators who prefer not to follow the above script.

- *Share a word, phrase, or what you wrote.*
- *What did you see or hear? How did it feel?*
- *Did anything in what you heard remind you of anything?]*

Concluding Remarks (Course Instructor to conclude; four minutes)

Thank you all for participating in today's Narrative Medicine Session using art as our inspiration. We hope that this will be the first of a more regularly scheduled experience for fellows to be able to participate in narrative-based practice together. As we take a moment, I invite you all to close your eyes and notice in your body any sensations, tightness, or breathing patterns. Notice if you feel your teeth unclench, your shoulders slightly straighter in your chair, and notice the feeling of your feet against the ground. Focus on your breathing as you breathe in and out. And when you are ready, open your eyes. Welcome back to our shared space. As we

close today's session, I invite you to share in the chat box a word or phrase that stood out to you today, or that you feel and will be taking home with you this evening.

We look forward to seeing you at our next session in just two weeks. Thank you for your time, your energy, and your presence, and if you are interested in participating in future narrative medicine-related work, we invite you to join our CARRA Narrative Medicine Workgroup. Please email the Course Instructor for more information!

Session II: Ambiguity, Uncertainty and Chance

1. **Part 1: Chance (25 min)**

Facilitator 1: Welcome back to our second of the four narrative medicine sessions. Unlike last time, today we are going to skip over the introductory lecture and jump right in. Before we start on our topic today, I invite you to close your eyes and notice in your body any sensations, tightness, or breathing patterns. Notice if you feel yourself clenching your teeth, slumping your shoulders, notice the feeling of your feet against the ground, of your back against the chair. Focus on your breathing as you breathe in and out. And when you are ready, open your eyes. Today, the first half of our session will open around chance. What are the odds that the patient was the one to have their diagnosis? Who decided to give this child a disease without a cure? We will start by having one person read this poem by Wislawa Szymborska, a winner of the 1996 Nobel Prize for Literature. Then we will have a second reader read the poem. Afterward, we will share the writing prompt, and you will have five minutes to write. We will let you know when you have one minute left. Who would like to read first? *[When there is one minute left, the facilitator asks: What else is also true?]*

[Below is a list of relevant points for facilitators who prefer not to follow the above script.

- *Welcome back*
- *Close your eyes, notice body sensations, focus on your breathing, then open your eyes*
- *Our first half today will focus on chance.*
- *Today's poem is by Wislawa Szymborska, a winner of the 1996 Nobel Prize for Literature*
- *Two participants will read the poem, then have five minutes to write*
- *We will let you know when you have 1 min left. (When there is one minute left, the facilitator asks: What else is also true?)]*

"Could Have"
By Wislawa Szymborska

It could have happened.
It had to happen.
It happened earlier. Later.
Nearer. Father off.
It happened, but not to you.

You were saved because you were the first.

You were saved because you were the last.
Alone. With others.
On the right. The left.
Because it was raining. Because of the shade.
Because the day was sunny.

You were in luck—there was a forest.
You were in luck—there were no trees.
You were in luck—a rake, a hook, a beam, a brake,
a jamb, a turn, a quarter inch, an instant.

As a result, because, although, despite.
What would have happened if a hand, a foot,
within an inch, a hairsbreadth from
an unfortunate coincidence.

So you are here? Still dizzy from another dodge, close shave,
reprieve?
One hole in the net and you slipped through?
I could not be more shocked or speechless.
Listen,
how your heart pounds inside me.

Facilitator 1: Now it is time to share. Who would like to share a word, phrase, or what they

wrote? What did you see or hear? How did it feel? Did anything in what you heard remind you of anything?

[Below is a list of relevant points for facilitators who prefer not to follow the above script.

- *Share a word, phrase, or what you wrote.*
- *What did you see or hear? How did it feel?*
- *Did anything in what you heard remind you of anything?]*

2. **Part 2: Ambiguity and Uncertainty (30 min)**

Facilitator 2: For the second half of our session today, we will focus on the ambiguity and uncertainty we deal with so frequently in medicine and in rheumatology in particular. Ambiguity is defined by the Merriam–Webster dictionary as uncertainty, or the quality or state of being doubtful or uncertain, especially because of obscurity or indistinctness. In pediatric rheumatology, we frequently face ambiguity, whether it be in making a diagnosis or deciding on treatment. In the arts, uncertainty or ambiguity is often embraced as a creative opportunity, and some argue that this artistic embrace of uncertainty can cast a positive outlook on the role uncertainty can have in the medical realm.

[Below is a list of relevant points for facilitators who prefer not to follow the above script.

- *The second half of today is focused on ambiguity and uncertainty*
- *Merriam–Webster's definition of ambiguity: "uncertainty, or the quality or state of being doubtful or uncertain especially because of obscurity or indistinctness"*

- *Pediatric rheumatology faces ambiguity frequently with diagnosis/treatment*
- *In art, uncertainty can be seen as a creative outlet and a good thing.*

Today, we face ambiguity in the form of this video, hearing from professional poker player Annie Duke, who will discuss Uncertainty as a hidden strength. We will watch the video together, and then spend five minutes writing about <u>What do not you know?</u> OR <u>What are you confident about?</u> We will let you know when you have one minute left, and then we will reconvene to share. *[When there is one minute left, the facilitator asks: What else is also true?]*

[Below is a list of relevant points for facilitators who prefer not to follow the above script.

- *We will hear from a professional poker player, Annie Duke, speak about uncertainty*
- *Writing prompts: What do you not know? OR What are you confident about?*
- *You will have five minutes to write; we will let you know when you have one minute left. (When there is one minute left, facilitator asks: What else is also true?)]*

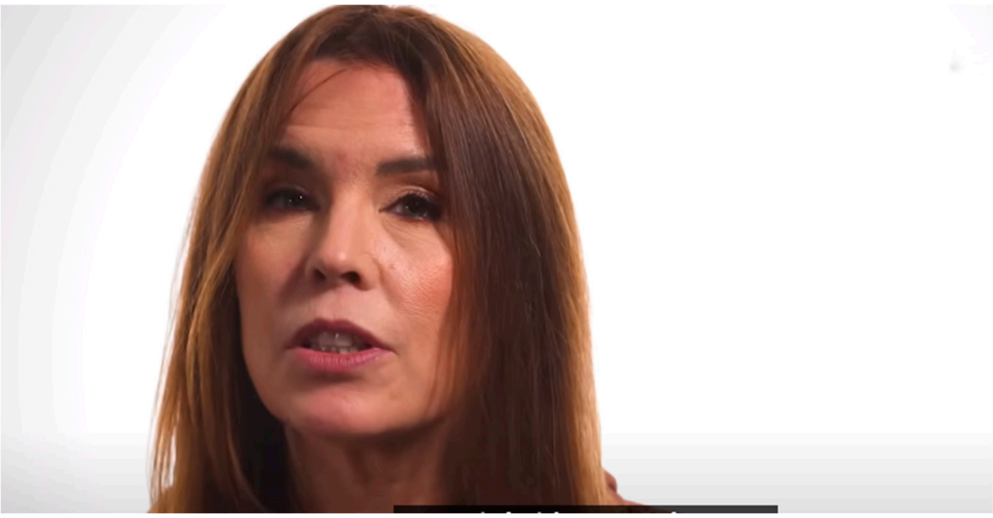

https://www.youtube.com/watch?v=ygJ1g1F3dfo

<u>Facilitator 2</u>: Now it is time to share. Who would like to share a word, phrase, or what they
wrote? What did you see or hear? How did it feel? Did anything in what you heard remind
you of anything?

[Below is a list of relevant points for facilitators who prefer not to follow the above script.

- *Share a word, phrase, or what you wrote.*
- *What did you see or hear? How did it feel?*
- *Did anything in what you heard remind you of anything?]*

Concluding Remarks (five minutes)

<u>Facilitator 1</u>: Thank you all for participating in today's Narrative Medicine. As we close today's session, I invite you to share in the

chat box a word or phrase that stood out to you today, or that you feel and will be taking home with you this evening. As we close our session, I invite you to close your eyes and notice in your body any sensations, tightness, or breathing patterns. Notice if you feel yourself clenching your teeth, slumping your shoulders, notice the feeling of your feet against the ground, of your back against the chair. Focus on your breathing as you breathe in and out. And when you are ready, open your eyes.

Thank you for your time, your energy, and your presence, and we will see you next time!

[Below is a list of relevant points for facilitators who prefer not to follow the above script.

- *Thank you for participating!*
- *Share a word/phrase that stood out to you or something you will be taking home with you in the chat box*
- *Close your eyes, notice body sensations, focus on your breathing, then open your eyes*
- *Thank you for your time, energy, and presence*

Videos to be sent to participants afterward:

Uncertainty with Kids: https://www.youtube.com/watch?v=Z2AWxGy0j8s

Coping with Uncertainty: https://www.youtube.com/watch?v=S3CU2kOBt3s

Session III: The Wounded Healer

Part I: The Wounded Healer: (25 min)

<u>Facilitator 1</u>: Welcome back, all. It is wonderful to see your faces and to be able to join together today. And now to introduce our topic for the day, the Wounded Healer. Some believe it is our woundedness that teaches us to be gentle with the wounds of others, to trust the natural and mysterious process of healing, and to develop an innate compassion and empathy for others. This can allow those in pain to not be alone when they are with us, and know that they are safe with us. This concept—that our wounds do not diminish us, they make us trustworthy—has proven to be a very healing idea for many individuals who fear that they are not perfect enough or strong enough to be able to help others. The Wounded Healer emerges in many of the world's oldest cultures and traditions of healing. The shamans or medicine men were often people who had an unhealed wound that they exhibited publicly as their credential of the wisdom to do healing work. These cultures accepted that it was the knowledge of the process of healing that comes from wounding, and the wisdom gained from the experience of wounding, that makes someone into a truly powerful healer.

Today, we will be writing about the Wounded Healer. Take a look at these two pieces and choose the artwork that speaks to you. On the left, you will see a sculpture from 2014 entitled 'The Wounded Healer,' by Marija Gauci, an art psychotherapist and visual artist born in Malta and based in the UK. On the right, you will see a

painting from 1939 entitled 'The Two Fridas' by Frida Kahlo, a Mexican painter known for her portraits and work inspired by nature and artifacts of Mexico. Think about what you notice about the photos in front of you. Which of these images strikes you the most—let us choose that one for your prompt. What words come to mind? Write down descriptions, colors, textures, a feeling, a thought, or anything that comes to you right away; you can write as we talk together here and during the five-minute exercise if desired. Our prompt tonight will have you choose one of these two photos and write for five minutes. If you choose the image on the left, write from the perspective of the sculptured individual OR from the perspective of the wound. If you choose the image on the right, write from the perspective of Frida. You can write a paragraph, a poem, or a story; it can make sense or be nonsensical, you do not have to worry about it. Just keep your hand or pencil moving; if you get stuck, keep writing the last word over and over until the next idea or thought comes to you. At the end of the five minutes, we will ask everyone to split into their breakout groups to discuss their writings. The prompts will be written in the chat for you to reference as well." *[Write the prompt (underlined above) in the chat.]*

[Below is a list of relevant points for facilitators who prefer not to follow the above script.

- *Welcome back!*
- *Close your eyes, notice body sensations, focus on your breathing, then open your eyes*
- *Today's topic is the Wounded Healer.*
  - *Our wounds do not diminish us; they make us trustworthy*
  - *Wounded healers can help others despite fear that they are not perfect enough or strong enough*
  - *Wounded healer is in some of the world's oldest cultures/traditions of healing. Shamans/medicine men were often people who had un-healable wound, which they exhibited publicly as their credential of wisdom to do healing work*
  - *Today's prompts: The Wounded Healer by Marija Gauci and The Two Fridas by Frida Kahlo*
    - *Choose the image that strikes you the most*
    - *Writing prompt for Marija Gauci: Write from the perspective of the sculptured individual OR from the perspective of the wound*
    - *Writing prompt for Frida: Write from the perspective of Frida*
    - *Write for five minutes. When there is one minute left, the facilitator asks: What else is also true?]*

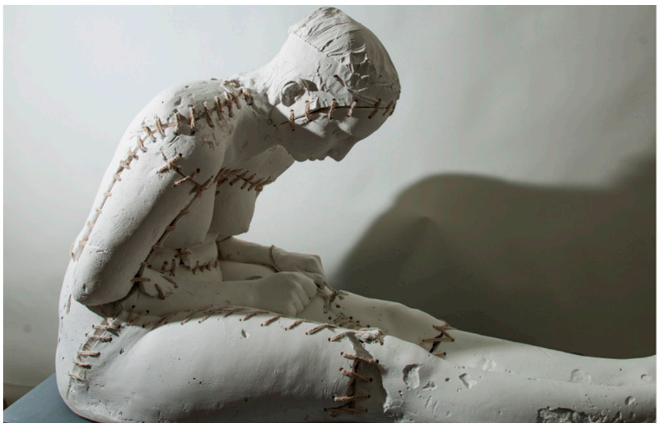
The Wounded Healer by Marija Gauci

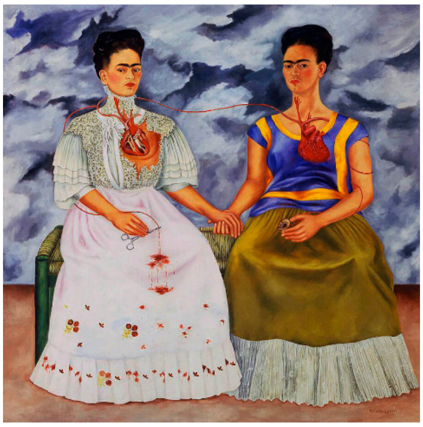
The Two Fridas, by Frida Kahlo

1. **Part II**: **Prompt Sharing** (20 min)

Facilitator 2: It is time to have space to share. It can be very scary to share your work, and that is okay; we are here to support each other through the process. As a reminder, when we respond to other people's work or words, try to say how it made you feel and avoid judgments. Tell us which image you chose, and you can choose a couple of images, adjectives, or a phrase. We have 10–12 min to share everyone's work. We will go around the room, and each person can say a word or two or a sentence from their piece, and if you do not want to share, you can say pass. We can go around again at the end if you feel comfortable sharing as well. Does anyone have a response to something they heard?

If there is extra time and folks are interested, offer a second writing prompt (five to seven minutes to write, remainder of time to share): write about a wound. OR Write about being stitched back together." *[Write prompt (underlined) in the chat.]*

[Below is a list of relevant points for facilitators who prefer not to follow the above script.

- *Share a word, phrase, or what you wrote.*
- *What did you see or hear? How did it feel?*
- *Did anything in what you heard remind you of anything?*
- *If there is extra time, second writing prompt x five to seven minutes (depending on time left): Write about a wound OR write about being stitched back together]*

Concluding Remarks (five minutes)

Facilitator 1: Thank you all for participating in today's Narrative Medicine Session using art as our inspiration. As we close today's session, I invite you to share in the chat box a word or phrase that stood out to you today, or that you feel and will be taking home with you this evening. I invite you to close your eyes and notice in your body any sensations, tightness, or breathing patterns. Notice if you feel yourself clenching your teeth, slumping your shoulders, notice the feeling of your feet against the ground, of your back against the chair. Focus on your breathing as you breathe in and out. And when you are ready, open your eyes.

We look forward to seeing you at our next session in just a few months. Thank you for your time, your energy, and your presence, and if you are interested in participating in future narrative medicine-related work, we invite you to join our CARRA Narrative Medicine Workgroup. Please email the Course Instructor for more information.

[Below is a list of relevant points for facilitators who prefer not to follow the above script.

- *Thank you for participating!*
- *Share a word/phrase that stood out to you or something you'll be taking home with you in the chat box*
- *Close your eyes, notice body sensations, focus on your breathing, then open your eyes*
- *Thank you for your time, energy, and presence*

Session IV: Allowing Awe in Medicine

Part I: Allowing Awe in Medicine (25 min)

Facilitator 1: Today, our theme is Allowing Awe in Medicine. Before we start our session, I invite you to close your eyes and notice in your body any sensations, tightness, or breathing patterns. Notice if you feel yourself clenching your teeth, slumping your shoulders, and notice the feeling of your feet against the ground, of your back against the chair. Focus on your breathing as you breathe in and out. And when you are ready, open your eyes.

Formal definitions of awe include words like respect, reverence, admiration, fear, and surprise regarding something powerful or sacred. The medical field is filled with experiences that may elicit a sense of awe and wonder, and for some, serve as one of the reasons to even enter medicine in the first place. One might feel wonder at the skill and wisdom of colleagues, admire the masterly actions of a mentor, respect the bravery and courage of a patient, or sit in awe in the face of manifestations and effects of disease or disease treatment. Emerging evidence suggests that experiences of awe and wonder can result in health-related benefits. Two studies that surveyed healthy freshmen undergraduates showed that awe, joy, contentment, and pride predict lower levels of interleukin-6 (IL-6), a pro-inflammatory cytokine, and dispositional awe had the strongest relationship with IL-6 of any positive emotion. [1,2]

Awe often begins with a story, and today, we will share the story of Judith and Joyce Scott, twins born in 1943 in Cincinnati, Ohio, to a middle-class family. (*Share screen with photo of Judith and Joyce in PowerPoint.*) Joyce was a healthy baby; her sister Judith was born with an extra chromosome and was diagnosed with Down syndrome. While they started their journey together, playing in the same space, sleeping in the same bed, and doing everything together, as they grew older, differences in their development began to manifest. Joyce began speaking; Judith did not. Many years later, it was discovered that Judith was left profoundly deaf by scarlet fever in infancy. As Judith's deafness went undiagnosed, her communicative skills were stunted, and she failed to qualify for the only public school class for children with learning

disabilities. And as time went on, in a world where it was practically unheard of for children with severe disabilities to live with their parents, Joyce, a seven-and-a-half-year-old, woke up one day in autumn 1950 to find her sister missing. Judith spent her years as a ward of the state of Ohio, and as Joyce grew into her own, she began visiting Judith at the institution and eventually became Judith's legal guardian. Joyce enrolled her sister in the Creative Growth Art Center in Oakland, a visionary arts center where individuals with mental or psychological difficulties are given total artistic freedom. It was not until Judith was introduced to fiber art through this program that she found her voice. She spontaneously started wrapping pieces of wood in fiber, fabric, and threads, and created totems, and as she continued with the art, she began experimenting with clothing and accessories on a larger scale. She even became the first individual with Down syndrome to be featured in the San Francisco Museum of Modern Art.[3]

For our prompt today, we will take a look at some of Judith's work. Looking at these five images, <u>which image are you most drawn to? What about it speaks most strongly to you? Write about what draws you to it, what words come to mind. Is there a story that emerges from the image?</u> <u>Are there shapes, colors, or textures that stand out to you?</u> Take five minutes, and we will let you know when you have one minute left.

[Below is a list of relevant points for facilitators who prefer not to follow the above script.

- *Welcome back!*
- *Close your eyes, notice body sensations, focus on your breathing, then open your eyes*
- *Today's topic is Allowing Awe in Medicine.*
    - *Formally define Awe with words like respect, reverence, admiration, fear, surprise, regarding something powerful or sacred*
    - *The medical field has many experiences that can bring about awe/wonder*
    - *Awe may inspire people to enter medicine in the first place*
        - *Feel wonder at the skill and wisdom of colleagues*
        - *Admire the masterly actions of a mentor*
        - *Respect the bravery/courage of a patient*
        - *Sit in awe of manifestations and effects of disease or disease treatment*
    - *Awe can result in health benefits*
        - *Two studies of healthy freshman undergraduates showed that awe, joy, contentment, and pride predict lower levels of IL-6 (pro-inflammatory cytokine)*
    - *Awe begins with a story: Judith and Joyce Scott (twins born in Cincinnati, OH, in 1943)*
        - *Joyce: healthy baby*
        - *Judith: Born with Down's syndrome, showed developmental delay and was found to be profoundly deaf following scarlet fever in infancy. Stunted*

    communication skills meant that she did not qualify for public school classes. At the age of seven-and-a-half, she was sent away to an institution.
    - *Joyce eventually learned of Judith's institutionalization and became her legal guardian, enrolling her in the Creative Growth Art Center in Oakland,* CA.
    - *At the creative arts program, Judith learned of fiber art and found her voice. Created totems, wrapped wood in fiber, fabric, and threads, and experimented with clothes/accessories. Became the first individual with Down Syndrome featured in the San Francisco Museum of Modern Art*
- *Today's prompt: Look at Judith's work.*
    - *Which image are you most drawn to? What about it speaks most strongly to you? Write about what draws you to it, what words come to mind. Is there a story that emerges from the image? Are there shapes, colors, or textures that stand out to you?*
    - *Take five minutes; we will let you know when you have one minute left. (When there is one minute left, the facilitator asks: What else is also true?)]*

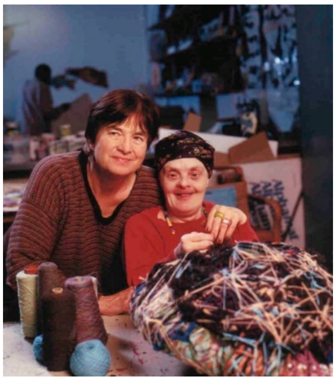

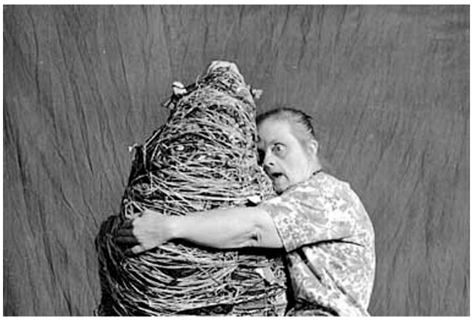

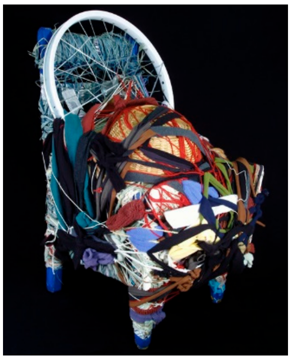

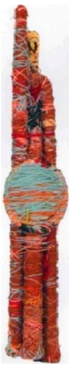

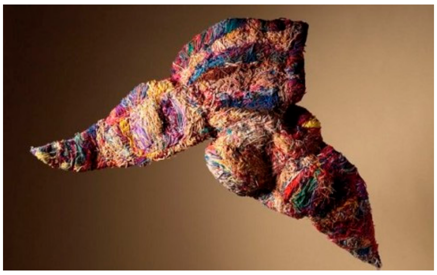

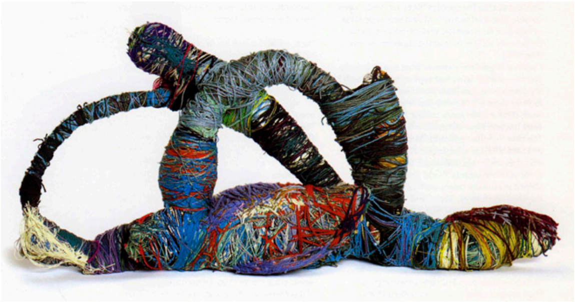

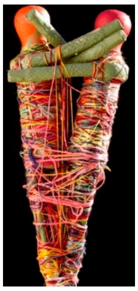

1. **Part II**: **Prompt Sharing:** Awe in Medicine (15 min)

 Facilitator 2: Now it is time to share. Who would like to share a word, phrase, or what they wrote? What did you see or hear? How did it feel? Did anything in what you heard remind you of anything?

 Thank you all for your thoughts and for sharing. This example of using our senses in a different way is a powerful example of the wonder in the world around us, with a person using the tools they may not have had otherwise to express their emotions and experiences. As we think about awe and wonder in medicine, it is important to bring this recognition of awe to our own practice. As we mentioned, cultivating and maintaining a sense of wonder can have health benefits and is essential to overcoming the routine and tedious aspects that can drive burnout in our practice. Our second prompt today is to take five minutes to write about a time in your professional or personal life when you had a sense of mystery or awe. What, if any, questions did it engender in you? You can write about your own experience, a colleague's, or something you observed years ago or this morning that continues to work on you. Perhaps there are questions related to this experience that continue to work on you. We will let you know when you have one minute left.

 Now it is time to share. Who would like to share a word, phrase, or what they wrote?

[Below is a list of relevant points for facilitators who prefer not to follow the above script.

- *Share a word, phrase, or what you wrote.*
- *What did you see or hear? How did it feel?*
- *Did anything in what you heard remind you of anything?*
- *If there is extra time, second writing prompt x five to seven minutes (depending on time left) to bring recognition of awe into our own practice: Write about a time in your professional or personal life that you had a sense of mystery or awe. What, if any, questions did it engender in you?*
  - *It is okay to write about a colleague's experience or your own experiences*
  - *(When there is one minute left for writing, the facilitator asks: What else is also true?)]*

2. **Part III: Reflections** (15 min)

Facilitator 1: As our session comes to a close, it is crazy to realize this is the last of our sessions together. With the time we have left, we want to open up the floor to hear comments/feedback on your experience during these sessions. What went well? What could be improved? What was your favorite or least favorite session?

Concluding Remarks (five minutes)

Facilitator 1: Thank you all for participating in today's Narrative Medicine Session using art as our inspiration. As we close today's session, I invite you to share in the chat box a word or phrase that stood out to you today, or that you feel and will be taking home with you this evening. I invite you to close your eyes and notice in your body any sensations, tightness, or breathing patterns. Notice if you feel yourself clenching your teeth, slumping your shoulders, and notice the feeling of your feet against the ground, of your back against the chair. Focus on your breathing as you breathe in and out. And when you're ready, open your eyes.

We look forward to seeing you at our next session in just a few months. Thank you for your time, your energy, and your presence, and if you are interested in participating in future narrative medicine-related work, we invite you to join our CARRA Narrative Medicine Workgroup. Please email the Course Instructor for more information.

[Below is a list of relevant points for facilitators who prefer not to follow the above script.

- *Thank you for participating!*
- *Share a word/phrase that stood out to you or something you'll be taking home with you in the chat box*
- *Close your eyes, notice body sensations, focus on your breathing, then open your eyes*
- *Thank you for your time, energy, and presence*

References:

1. Hart J. Awe and wonder in medicine. *Alt Complement Ther* 2018;24(4). DOI: 10.1089/act2018.29175.jha.
2. Stellar JE, John-Henderson N, Anderson CL, Gordon AM, McNeil GD, Keltner D. Positive affect and markers of inflammation: discrete positive emotions predict lower levels of inflammatory cytokines. *Emotion.* 2015;15(2):129-133.
3. https://www.textileartist.org/textile-artist-judith-scott-uncovering-innate-talent/
